# Autism and mental retardation with convulsion in tuberous sclerosis: a case report

**DOI:** 10.4076/1757-1626-2-7061

**Published:** 2009-07-31

**Authors:** Asok Kumar Datta, Syamali Mandal, Suvo Bhattacharya

**Affiliations:** 1 Department of Pediatrics Medicine, Burdwan Medical College and HospitalBurdwan, West BengalIndia; 2 Department of Gynecology and Obstetrics, Burdwan Medical College and HospitalBurdwan, West BengalIndia

## Abstract

A 6-year-old male child born of a non-consanguineous marriage admitted in the pediatrics emergency ward with the history of recurrent attacks of convulsion since 4 month of age. He was also suffering from frequent vomiting. Examination revealed that the child had characteristics features of angiofibromas on the face with butterfly distribution, hyperpigmented patches on forehead, hypopigmented macules on trunk, prominent subependymal and cortical tubers. The child was diagnosed as tuberous sclerosis. Association of autistic behaviors and severe degree of mental retardation are noteworthy in this child indicating the need of counseling as early as possible along with behavioral and educational strategies for mental retardation from early age with other symptomatic management.

## Introduction

Tuberous sclerosis (TSC) has an incidence of 1 in 6000 to 1 in 10000 live births with no ethnic clustering [1]. TSC is an autosomal dominant disease. Genetic studies detected two loci e.g. TSC1, the abnormality is located on chromosome 9q34 and TSC2, the abnormality is located on chromosome 16p13. Approximately two thirds of cases are sporadic that is, affected individuals have no family history of the disease [2].

TSC1 gene encodes tuberin and the TSC2 gene encodes hamartin. Hamartin and tuberin form a complex that is thought to negatively regulate the cell cycle. TSC results from mutations in the TSC1 (hamartin) and TSC2 (tuberin) genes [3]. The presence of either mutation produces uncontrolled proliferation and differentiation in numerous tissues including the skin, central nervous system (CNS), heart, skeleton and kidneys. CNS hamartomas can cause seizures, mental retardation and autism.

TSC is an extremely heterogeneous disease with a wide clinical spectrum varying from severe mental retardation and incapacitating seizures to normal intelligence and a lack of seizures often within the same family.

## Case presentation

A 6-year-old male child born of a non-consanguineous marriage from a middle class family of a rural area of Burdwan admitted in the emergency pediatrics ward with the history of convulsion associated with frequent vomiting. According to the mother the problem had started since 4^th^ month of age which was initially more on the right side gradually progressing to affect whole of the body. The vomiting occurred frequently with or without convulsion.

The child was developmentally lacking behind other children of the same age and sex of the community. He was not able to speak and communicate till now, very much sleepy and did abnormal behavior frequently.

Mother noticed changes in the skin since 4th year of age, there were multiple tiny pink nodules on the nose and malar prominences which later coalesced to form fleshy appearances. There are hyperpigmented patches on left forehead and right malar regions. Patchy hypopigmented areas were present on both front and back of the trunk.

There was no other member of the family suffering from the disease.

**Figure 1.**
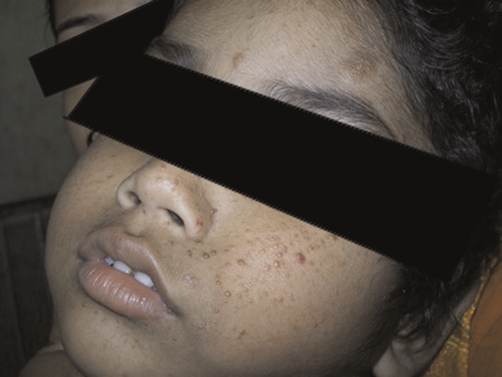
Shows the characteristics skin lesions on the face. There are profuse pinkish papulo nodular lesions which in some places coalesce to form fleshy lesion distributed over the nose and both malar regions. Small lesions are also present in other areas also. Notifiable important one large hyperpigmented lesion on left forehead is also there.

On examination, the child was found dull, indifferent and expressionless with an average built having weight 20 kg, height 122 cm, head circumference 52 cm and chest circumference 61 cm. There were extensive facial angiofibromatosis with butterfly distribution covering nose and spreading to cheeks (Figure 1), two prominent hyperpigmented plaques were seen, one on left forehead covering a wide area (Figure 1) and one on right cheek. There were hypopigmented patches abundant on both sides of the trunk.

The child had disturbances of higher function like orientation in time and place, speech and language as well as intelligence. His cranial nerves and motor functions were normal. Other neurological and systemic examinations appeared to be normal.

Psychiatric assessment with the help of Vineland Social Maturity Scale and Conner’s Autism Rating Scale revealed the child was suffering from autism along with severe mental retardation with intelligent score was about 25 to 30.

**Figure 2.**
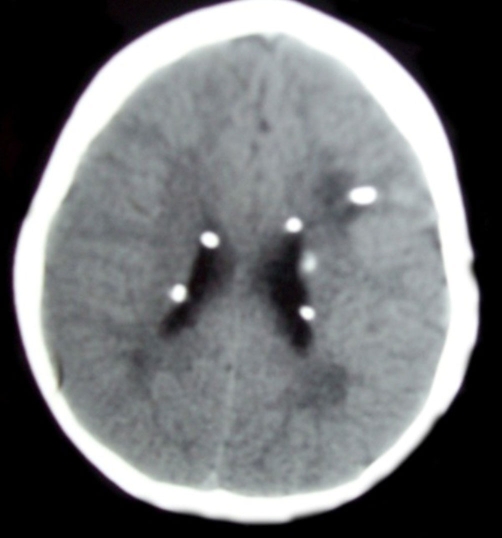
CT scan of the brain showed multiple foci of Subependymal calcification looked like candle-dripping appearances; they were also present in both basal ganglia, both temporal and left parietal regions.

C T scan of the brain showed multiple foci of Subependymal calcification looked like candle-dripping appearances; they were also present in both basal ganglia, both temporal and left parietal regions (Figure 2).

Ultrasonography of kidneys and liver showed no abnormality, Echocardiography of heart revealed no rhabdomyoma of cardiac muscle. Opthalmolscopic examination was also normal.

The child was treated with higher doses of sodium valproate and the convulsion was controlled. He was referred to the Psychiatric department for necessary measures.

## Discussion

In 1998, the National Institutes of Health convened a consensus conference to standardize diagnostic criteria for the TS [4]. The published set of criteria was composed of clinical and radiographic features, which were divided into major and minor categories. A definitive diagnosis of TS requires that a patient present with two of the major criteria shown in Table 1 or one major and two minor criteria. No single criterion, found either clinically or radiographically, is present in all patients.

**Table 1.** Diagnostic criteria for tuberous sclerosis

Major Features	Minor Features
facial angiofibromas	multiple pits in dental enamel
ungual or periungual fibroma	hamartomatous rectal polyps
hypomelanotic macules	bone cysts
shagreen patch	“migration tracts”
cortical tuber	gingival fibroma
Subependymal nodule	nonrenal hamartoma
Subependymal giant cell astrocytoma	retinal achromic patch
multiple retinal nodular hamartomas	“confetti” skin lesions
cardiac rhabdomyoma	multiple renal cysts
lymphangiomyomatosis	
renal angiomyolipoma	

The most frequently observed manifestations are those of the skin and of the central nervous system like seizures, mental retardation, followed by renal, cardiac and ocular manifestations. Among cutaneous manifestations, hypomelanotic macules, facial angiofibromas, shagreen spots, fibrous plaques on the forehead and ungula fibroma are observed [5].

There is a statistically significant relationship between the presence of a forehead plaque and CNS involvement in TSC. Therefore, forehead plaque may be considered as a novel cutaneous marker to know the CNS involvement in TSC at an early stage [6].

In our patient, we had hypomelanotic macules present in large number on the front and back of the trunk, facial angiofibromas lesions were present on the face over nose and malar region in butterfly distribution (Figure 1), hyper pigmented patches on left upper forehead and right cheek were present.

Tubers were present throughout the cortex and mostly in subependymal regions which may give rise to candle- dripping appearance (Figure 2). Sometimes the tuber converts to giant cell astrocytoma which may block the foramen of Monro resulting in hydrocephalus. Due to cortical tubers the convulsion is a most common and early feature of this disease. Any type of convulsion from infantile spasm, myoclonic convulsion to persistent tonic convulsion may occur.

Our patient presented with convulsion as the first manifestation at the age of 4 months and admitted this time with severe intractable convulsion.

Systematic evaluation of neuropsychological attention skills in a population-derived sample of children and adolescents with TSC showed that, even when age, gender, IQ, and intra-familial clustering were controlled for, the TSC group had significantly lower scores than their unaffected siblings on a range of neuropsychological attentional tasks, and that they had significantly more neuropsychological attention deficits. The findings suggest that clinical neuropsychological evaluation of attentional skills should be performed in children and adolescents with TSC [7].

Psychometric analysis of our patient revealed poor adjustment and eye contacts, playing ability was poor, hyperactivity, autistic features, impaired speech and language. Total data suggested moderately autistic child with mental retardation. Intelligent quotient was found to be 25-30.

Other systems like ocular manifestations of hypopigmented macule on iris, retinal phakomas and renal angiomyolipomas are more common in older age group. Cardiac rhabdomyomas may present in almost of half of the pediatric cases.

Treatment is symptomatic. Anticonvulsants for seizures, shunting for hydrocephalus, and behavioral and educational strategies for mental retardation are the mainstays of management. The mainstay of seizure control for patients with TS is medical therapy with anticonvulsant drugs and a ketogenic diet [8]. Evidence is accumulating that vigabatrin, an inhibitor of γ-aminobutyric acid transaminase, is the anticonvulsant medication of choice for patients with TS [9].

Vigabatrin is not available in our set up. We have controlled the seizure with sodium valproate.

In one study, the authors present a 10-year-old girl with tuberous sclerosis complex who has been receiving rapamycin for 10 months for seizure control. There was a dramatic reduction in seizure frequency with rapamycin therapy [10].

Rapamycin is still an experimental drug and is not available for pediatric use in India. If anticonvulsant medications and dietary modifications are not effective, then neurosurgical intervention can be considered in selective cases.

## Conclusion

In our case we found the fulfillment of the diagnostic criteria of TSC. The autism and mental retardations are important association, which everybody should look for. Along with the treatment with anticonvulsive drugs regular counseling as early as possible should be done along with behavioral and educational strategies for mental retardation.

## Abbreviations

CNS: central nervous system
CT: computed tomography
TSC: tuberous sclerosis
